# Predator‐prey feedback in a gyrfalcon‐ptarmigan system?

**DOI:** 10.1002/ece3.4563

**Published:** 2018-11-28

**Authors:** Frédéric Barraquand, Ólafur K. Nielsen

**Affiliations:** ^1^ CNRS Institute of Mathematics of Bordeaux Talence France; ^2^ Integrative and Theoretical Ecology, LabEx COTE University of Bordeaux Pessac France; ^3^ Icelandic Institute of Natural History Garðabær Iceland

**Keywords:** consumer‐resource, *Falco rusticolus*, *Lagopus muta*, MAR, population cycles, VAR

## Abstract

Specialist predators with oscillating dynamics are often strongly affected by the population dynamics of their prey, yet they are not always the cause of prey cycling. Only those that exert strong (delayed) regulation of their prey can be. Inferring predator–prey coupling from time series therefore requires contrasting models with top‐down versus bottom‐up predator–prey dynamics. We study here the joint dynamics of population densities of the Icelandic gyrfalcon *Falco rusticolus*, and its prey, the rock ptarmigan *Lagopus muta*. The dynamics of both species are likely not only linked to each other but also to stochastic weather variables acting as confounding factors. We infer the degree of coupling between populations, as well as forcing by abiotic variables, using multivariate autoregressive models MAR(p), with *p* = 1 and 2 time lags. MAR(2) models, allowing for species to cycle independently from each other, further suggest alternative scenarios where a cyclic prey influences its predator but not the other way around (i.e., bottom‐up scenarios). The classical MAR(1) model predicts that the time series exhibit predator–prey feedback (i.e., reciprocal dynamic influence between prey and predator), and that weather effects are weak and only affecting the gyrfalcon population. Bottom‐up MAR(2) models produced a better fit but less realistic cross‐correlation patterns. Simulations of MAR(1) and MAR(2) models further demonstrate that the top‐down MAR(1) models are more likely to be misidentified as bottom‐up dynamics than vice versa. We therefore conclude that predator–prey feedback in the gyrfalcon–ptarmigan system is likely the main cause of observed oscillations, though bottom‐up dynamics cannot yet be excluded with certainty. Overall, we showed how to make more out of ecological time series by using simulations to gauge the quality of model identification, and paved the way for more mechanistic modeling of this system by narrowing the set of important biotic and abiotic drivers.

## 1 INTRODUCTION

Theoretical ecology predicts that among predators, specialists are the most likely to shape the dynamics of their prey (e.g. Andersson & Erlinge, 1977; Gilg, Hanski, & Sittler, 2003; Turchin & Hanski, 1997). It has even been suggested that only specialist predators do exhibit multi‐generation predator–prey population cycles (Murdoch et al., 2002), based on cycle periods in specialist versus generalist predators. Mechanistic modeling, however, disputes this particular point (Erbach, Lutscher, & Seo, 2013) Testing more thoroughly this working theory with empirical data — the more specialized the predator, the higher the likelihood of a predator–prey cycle or more generally top‐down prey regulation — would require to estimate the strength of predator–prey coupling in a number of real predator–prey systems, for which time series of both predator(s) and prey are available, preferably in the field. While the task may appear straightforward in theory, it is surprisingly difficult in practice. Cases of long‐term monitoring including both specialized predators and their main prey, through extended periods of time, are indeed quite rare, especially in vertebrates. Two famous exceptions to the rule include the wolf–moose (*Canis lupus* ‐ *Alces Alces*) system of Isle Royale (Vucetich, Hebblewhite, Smith, & Peterson, 2011), that has been followed for a century (although this study area is somewhat restricted for such wide‐ranging species), and the celebrated cycle of the Canada snowshoe hare *Lepus americanus*, which interacts with the Canada lynx *Lynx canadensis* and other predators (Krebs, Boonstra, Boutin, & Sinclair, 2001; Vik, Brinch, Boutin, & Stenseth, 2008). While there is a convincing array of evidence showing that lynx has a dynamical impact on hare (Vik et al., 2008), and wolf has an impact on moose (Vucetich et al., 2011), there is also evidence that weather and other drivers have often a strong forcing influence on prey dynamics (Vucetich & Peterson, 2004; Yan, Stenseth, Krebs, & Zhang, 2013). Even in such strongly interacting systems that fascinate the imagination by demonstrating strong oscillations, it has been suggested that the presence of an ubiquitous external forcing hardly warrants to view such systems as a pair of autonomous coupled differential equations (Barraquand et al., 2017; Nisbet & Gurney, 1976), despite the pivotal role of autonomous and deterministic dynamical systems in ecological theory (see e.g., McCann, 2011). Rather, real predator–prey systems are constantly buffeted by outside forces, be those climatic or biotic variables unaccounted for (i.e., other players in the interaction web). The study of Vik et al. (2008) reports at best around 55% of prey variance in log‐densities explained by both prey and predator densities; this therefore leaves ample room for other factors to influence hare dynamics (Barraquand et al., 2017; see also Vucetich et al., 2011 on ungulate‐wolf systems). In birds, contrasted feedback structures (bottom‐up or top‐down) were found in goshawk (*Accipiter gentilis*)—grouse dynamics, depending on the grouse species considered (Tornberg et al., 2013), with marked effects of weather forces. To gain a better appraisal of the strength of top‐down regulation in the field, compared to other drivers of herbivore dynamics (see Sinclair, 2003, for a discussion in mammals), the list of predator–prey systems to which stochastic models of interacting populations are fitted to time series needs to increase.

Our goal here is to contribute, using large‐scale field data, to improving the understanding of predator–prey dynamics. We do this by fitting stochastic, statistically driven predator–prey models to a presumably tightly coupled predator–prey pair, gyrfalcon *Falco rusticolus* and rock ptarmigan *Lagopus muta* in North‐East (NE) Iceland. The gyrfalcon is a predator specialized on ptarmigan (rock ptarmigan and willow ptarmigan *Lagopus lagopus*) (Nielsen & Cade, 2017). In Iceland, the rock ptarmigan amounts to on average 72% by biomass of the gyrfalcon summer diet (range 52%–86%, Nielsen, 1999). Previous studies analyzed periodicities using cross‐correlation functions and autoregressive models on each species separately (Brynjarsdóttir, Lund, Magnússon, & Nielsen, 2003; Nielsen, 2011), highlighting possible reciprocal coupling between the two time series. Here, we combine detailed monitoring data with multivariate autoregressive (MAR) modeling that allows to examine both dynamical linkages between species and effects of weather variables (Hampton et al., 2013; Ives, Dennis, Cottingham, & Carpenter, 2003). MAR modeling has been largely developed in econometrics (Granger, 1969; Lütkepohl, 2005), where it is primarily used to establish causal relationships in the sense of prediction (i.e., a variable has causal influence if it helps improving predictions about the future, Granger, 1969), which is the statistical philosophy that we adopt here.

## 2 MATERIALS AND METHODS

### 2.1 Study area and design

The study area (5,327 km^2^) in NE Iceland and survey methods used have been extensively detailed elsewhere (Nielsen, 1999, 2011) so we will remain brief. The study area is centered on Lake Mývatn (N65°60′, W17°00′) and is constituted of hilly terrain up to 600–800 m above sea level. The gyrfalcon population is censused annually by visiting all known territories within the study area to determine predator occupancy (*n* = 83 territories). The number of territorial rock ptarmigan males is surveyed every spring (mostly in May) on six plots (total area 26.8 km^2^) within the general study area. The study started in 1981, and we used data for the period 1981–2014.

### 2.2 Ecological variables

We consider two main variables, the occupancy rate of gyrfalcon territories, that was considered a good proxy for gyrfalcon population density, and mean density of territorial rock ptarmigan cocks on the six plots. Both variables are standardized in the statistical models.

We also consider weather variables that are known to potentially affect the dynamics of the two populations. Higher temperatures are known to have positive effects on ptarmigan chick survival (Nielsen, Brynjarsdóttir, & Magnússon, 2004), and a previous study found that April mean temperature and precipitation affect all parameters of the gyrfalcon breeding success (Nielsen, 2011). We have selected three stations for the temperature (Akureyri, Mánárbakki, and Grímsstaðir) and six stations for log‐precipitation (Lerkihlíð, Mýri, Staðarhóll, Reykjahlíð, Mánárbakki, and Grímsstaðir), all within or at the border of the study area and that have recordings from 1975 to now. The weather data were retrieved from the web site of the Icelandic Met Office (www.vedur.is).

### 2.3 Statistical models

Multivariate Autoregressive (MAR) models have been used to assess the strength of predator–prey coupling (Ives et al., 2003; Vik et al., 2008). Let us denote the ln‐transformed predator density, *p*_t_ = ln(*P*_t_), and the ln‐transformed density of the prey, *n*_*t*_ = ln(*N*_*t*_); the log transformation is useful to transform log‐normal into Gaussian noise. These ln‐densities are then centered and stacked into a vector **x**_*t*_ = (*x*_1*t*_, *x*_2*t*_)′ = (*n*_*t*_, *p*_*t*_)′. The dynamics of the MAR(1) model, with one time lag, are then written as a forced recurrence equation (Equation (1)),

(1) $$x_{t+1}\;=\;a+{\mathrm{Bx}}_{t}+{\mathrm{Cu}}_{t}+e_{t},e_{t}∼N_{2}\left(0,\Sigma \right)$$

where **B** is an interaction matrix that characterizes the effects of net interactions on population growth of the two species, **C** describes the effect of environmental covariates **u**_*t*_ on the population growth rates of predator and prey, and **e**_*t*_ is a Gaussian bivariate noise term, with covariance matrix **Σ**. Throughout the text, boldface quantities represent vectors and capital bold, matrices.

The model with full interaction matrix has matrix $B\;=\;\left(\begin{array}{cc} b_{11} & b_{12}\\ b_{21} & b_{22} \end{array}\right)$. In this matrix, *b*_12_ represents the effect of the predator (species 2) log‐density on the prey (species 1) growth rate, while *b*_21_ represents the effect of the prey on the predator. *b*_11_ and *b*_22_ are the strengths of self‐regulation in the prey and predator, respectively, for more details see Ives et al. (2003). We also considered a model without interactions, where $B\;=\;\left(\begin{array}{cc} b_{11} & 0\\ 0 & b_{22} \end{array}\right)$, hereafter referred to as the null MAR(1) model. The diagonal **B** matrix essentially assumes independent AR(1) processes for the two species, forced by weather variables, provided that the variance–covariance matrix is also diagonal.

Both models were considered without (**C** = 0) and with environmental forcing (**C** ≠ 0). The weather variables that we stacked within **u**_*t*_ are delayed: The predator population is believed to be affected by weather 5 years before, because recruits enter the adult population at the age of 4 years (average time to maturity, Nielsen, 1990), while the prey population is affected by the weather of the year (between *t* and *t*+1) or that of the preceding year (between *t*−1 and *t*). We considered models with temperature effects, log(precipitation) effects, or both. The gyrfalcon population was assumed to be closed (despite wide‐ranging movements of other gyrfalcon populations, Burnham & Newton, 2011; reproductive birds are from Iceland), which is confirmed by genetic analyses (Johnson, Burnham, Burnham, & Mindell, 2007).

Several model fitting techniques have been considered in preliminary explorations (MCMC using JAGS within R, least squares for vector autoregressive models in R package vars, simple independent linear autoregressive models using lm() in R). Maximum‐likelihood estimation using the MARSS package (Holmes, Ward, & Wills, 2012) and the EM algorithm was finally chosen because it allowed to easily perform model selection for contrasted interaction matrices (i.e., setting some interactions to zero). All algorithms gave, however, similar model estimates (see Supporting Information Appendix S1).

We then considered more complex MAR(2) models that are able to allow for both populations to cycle independently, because each univariate AR(2) component can model long cycles (≈7–10‐year cycles, like those observed in the field). Selection of the optimal time lag *p* in MAR(p) model using a variety of model information theoretic criteria (see code in https://github.com/fbarraquand/GyrfalconPtarmigan_MAR) suggested an optimal lag order of 2 (BIC, HQ) or 3 (AIC, FPE). Because 2 time lags are enough to model independently cycling populations of period up to 10 years and more (Royama, 1992), and MAR(2) models are already parameter‐rich, we considered a maximum of 2 time lags in MAR models. The MAR(2) model can be written as

(2) $$x_{t+1}=B^{\left(1\right)}x_{t}+B^{\left(2\right)}x_{t-1}+{\mathrm{Cu}}_{t}+e_{t},e_{t}∼N_{2}\left(0,\Sigma \right)$$

where the two **B** ^(1)^ and **B** ^(2)^ matrices correspond to the two time lags. The independent cycling model has diagonal matrices **B** ^(1)^ and **B** ^(2)^. The full model has interaction matrices $B^{\left(1\right)}\;=\;\left(\begin{array}{cc} b_{11}^{\left(1\right)} & b_{12}^{\left(1\right)}\\ b_{21}^{\left(1\right)} & b_{22}^{\left(1\right)} \end{array}\right)$ and $B^{\left(2\right)}\;=\;\left(\begin{array}{cc} b_{11}^{\left(2\right)} & b_{12}^{\left(2\right)}\\ b_{21}^{\left(2\right)} & b_{22}^{\left(2\right)} \end{array}\right)$. To model also an asymmetric and nonreciprocal effect from the cyclic prey to its predator, we used the following interaction matrices $B^{\left(1\right)}\;=\;\left(\begin{array}{cc} b_{11}^{\left(1\right)} & 0\\ 0 & b_{22}^{\left(1\right)} \end{array}\right)$ and $B^{\left(2\right)}\;=\;\left(\begin{array}{cc} b_{11}^{\left(2\right)} & 0\\ b_{21}^{\left(2\right)} & b_{22}^{\left(2\right)} \end{array}\right)$. The model was named “bottom‐up”, in order to designate a predator dynamics driven by that of its cyclic prey.

When including time series *x* in a time series model for *y* improves the in‐sample prediction of *y* (i.e., reduces the residual variance), *x* is said to Granger‐causes *y* (Detto et al., 2012; Granger, 1969). The concept is therefore interwoven with nonzero interaction coefficients between system components (here, predator and prey). Granger causality testing was done with grangertest() using a Wald test, in the R package lmtest.

### 2.4 Evaluating the quality of model identification from simulated data

We used the above‐mentioned models, with fitted coefficients for the **B** ^(1)^ and **B** ^(2)^ matrices, to simulate dynamics for different time series lengths (*n* = 35 and *n* = 100 time steps). Specifically, we performed 1,000 simulations of the fitted MAR(1) full (F) model and MAR(2) bottom‐up (BU) model. We fitted the MAR(1) F model to both MAR(1) F and MAR(2) BU simulations. We then fitted the MAR(2) BU to both MAR(1) F and MAR(2) BU simulations. This allowed to compute the percentage of correctly ascribed scenarios, on the basis of information criteria (AIC, AICc, BIC) for each simulated model. This analysis is similar to the one performed by Lütkepohl (2005), which we report in the Discussion section.

We also simulated 100 times the fitted models to assess their ability to reproduce the observed cross‐correlation pattern between predator and prey.

## 3 RESULTS

### 3.1 MAR(1) model results

#### 3.1.1 Models without environmental covariates

**Figure 1:**
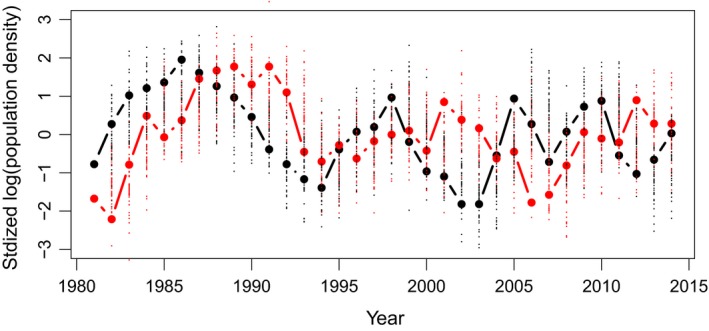
Time series of gyrfalcon (red) and rock ptarmigan (black) standardized log‐densities in NE Iceland, and their corresponding one step ahead predictions under the best‐fitted, full interaction matrix MAR(1) model. 100 model simulations one step ahead are plotted, for each year, as small points—red for predator and black for prey

The predator–prey time series and the MAR(1) model one step ahead predictions are presented in Figure 1, while Table 1 shows the MAR(1) model‐fitted parameters. All **B** coefficients are found to be significantly different from zero, with commensurate strengths of predator → prey (*b*_12_) and prey → predator (*b*_21_) interaction. There is therefore a consistently negative effect of predator on prey and a consistently positive effect of prey on predator. Note that a clear‐cut sign was not an obligatory outcome, given those are net interaction coefficients, blending several ecological processes (e.g., direct and indirect predation effects) into one number (Certain, Barraquand, & Gårdmark, 2018).

**Table 1:** Estimates of the MAR(1) full model without environmental covariates. The off‐diagonal interaction coefficients are at or near statistical significance at a 95% level. Similar results are obtained for non‐diagonal **Σ** (not shown); for parsimony, we use a diagonal error matrix

Parameters	Meaning	Value	*SE*	Lower 95% CI	Upper 95% CI
*b*_11_	Prey→prey	0.7710	0.1112	0.5528	0.9890
*b*_21_	Prey→predator	0.2150	0.1096	0.0001	0.4298
*b*_12_	Predator→prey	−0.2333	0.1114	−0.4516	−0.0149
*b*_22_	Predator→predator	0.6601	0.1097	0.4449	0.8752
$\sigma_{1}^{2}$	Noise var. prey	0.3961	0.0774	0.2280	0.6103
$\sigma_{2}^{2}$	Noise var. predator	0.3844	0.0763	0.2212	0.5922

Based on the comparison of AICc and BIC between the full 2 × 2 interaction matrix and the diagonal matrix model (null model), the full model was favored (Table 2). The model with environmental covariates did not lead to substantially better fit or very different biotic interaction parameters (Table 2) than the full model, and the weather effects were not consistent (Table 3), save for those of delayed April temperature on predator growth.

**Table 2:** Comparison of model selection criteria for MAR(1) models. MAR(1) “null’’ indicates a diagonal **B** matrix while MAR(1) “full’’ indicates a full 2 × 2 interaction matrix. Models including temperature effects on growth rates (third row and below) take the form $x_{t+1}=a+{\mathrm{Bx}}_{t}+{\mathrm{Cu}}_{t}+e_{t},e_{t}∼N_{2}\left(0,\Sigma \right)$. Here, the environmental vector is $u_{t}={\left(T_{t-l_{P}+1},R_{t-l_{P}+1},T_{t-l_{G}+1},R_{t-l_{G}+1}\right)}^{\prime }$, with *T* the temperature and *R* log‐precipitation. There is a time lag *l*_*P*_ for the ptarmigan (0 or 1 year) and *l*_*G*_ = 5 (always) for the gyrfalcon: Weather is expected to have such delayed effects on the gyrfalcon counts because of age structure. April weather is considered for gyrfalcon as it is the critical period for reproduction, and it is always included in models from row 3 and below. Models from rows 3 to 7 considered May temperature for ptarmigan, log(precipitation), or both. The models of rows 8 and 9 considered instead July and June temperatures as environmental variables for ptarmigan

Model type	LogLik.	AIC	AICc	BIC
MAR(1) null	−70.01	148.0	148.7	154.1
MAR(1) full	−66.14	144.3	145.7	153.4
MAR(1) full + May temperature year *t*+1	−63.98	144.0	146.4	156.2
MAR(1) null + May temperature year *t*+1	−67.03	146.1	147.4	155.2
MAR(1) full + May temp. of year *t*	−63.94	143.9	146.3	156.1
MAR(1) full + May log(precipitation) of *t*	−64.89	145.8	148.2	158.0
MAR(1) full + May temp + log(precipitation)	−62.81	145.6	149.5	160.9
MAR(1) full + July temperature year *t*	−61.95	143.9	147.8	159.2
MAR(1) full + June temperature year *t*	−63.79	147.6	151.4	162.8

**Table 3:** Coefficients for biotic and abiotic effects on population growth. Species 1 is ptarmigan, and species 2 is gyrfalcon. May variables only affect species 1 while April variables, delayed by 5 years (we model the effect of variables at *t*−4 on growth between *t* and *t*+1), affect only species 2's population growth

Parameters	Value	*SE*	Lower 95% CI	Upper 95% CI
*b*_11_	0.7475	0.1071	0.5376	0.9575
*b*_21_	0.2031	0.1044	−0.0014	0.4077
*b*_12_	−0.1984	0.1135	−0.4210	0.0241
*b*_22_	0.7021	0.1043	0.4977	0.9065
Temperature May_*t*+1_	−0.0853	0.1142	−0.3092	0.1385
Precipitation May_*t*+1_	−0.1596	0.1091	−0.3735	0.0541
Temperature April_*t*−4_	0.2072	0.1061	−0.0008	0.4153
Precipitation April_*t*−4_	−0.0558	0.1068	−0.2652	0.1535
$\sigma_{1}^{2}$	0.3653	0.0743	0.2104	0.5628
$\sigma_{2}^{2}$	0.3418	0.0733	0.1943	0.5307

Additional Granger causality testing using the MAR(1) model revealed a two‐way reciprocal feedback, although the Wald test was only weakly significant (at the 0.1 level) due to the low sample size (i.e., the number of time points is large by ecological standards but small for time series analysis). Accounting for this relative shortness of the time series when interpreting statistical significance, the MAR(1) model strongly suggests a reciprocal predator–prey coupling of the ptarmigan and gyrfalcon populations.

#### 3.1.2 Models with environmental covariates

The addition of environmental covariates did not improve significantly model fit (Table 2). The coefficients were mostly non‐significant, as illustrated by the model including both temperature and log(precipitation) (0 is included within CIs for environmental **C** matrix coefficients, Table 3). The model with both precipitation and temperature was deemed over‐parameterized by the information criteria. It is likely that an effect of 5‐year delayed temperature on predator growth is present as this effect was found positive, relatively large and nearly statistically significant at 95% (Table 3). However, this weather effect does not seem to improve much the predictive ability of the model.

The effect of temperature in May_*t*+1_ (May of the year) on ptarmigan growth, by contrast, is both not statistically different from zero and of unexpected sign (negative here, while positive temperature usually has positive effects on the ptarmigan chicks, Nielsen et al., 2004). The effect of rain in May_*t*+1_ on ptarmigan population growth was negative and relatively strong, but not statistically significant at 95% (point estimate −0.1596, 95% CI: [−0.3735; 0.0541]). It is therefore possible that such a negative effect is present, but it does not appear clearly with the current dataset.

We also fitted models where winter weather affects ptarmigan growth (Supporting Information Appendix S2), to test the idea that the survival of first‐year chicks might be lower in harsher winters, but again we did not find consistent effects of weather on ptarmigan dynamics.

### 3.2 MAR(2) model results

The MAR(2) models showed uniformly better fit than the MAR(1) models (Table 4). Note that, in order to make this comparison, we re‐fitted the MAR(1) model with one less year to compare MAR(1) and MAR(2) models with an equal number of points, as any difference in data can strongly affect AIC and BIC values. The MAR(2) model with independent populations (i.e., diagonal interaction matrices **B** ^(1)^ and **B** ^(2)^) and the bottom‐up predator–prey model (see Methods section), assuming an independently cycling prey and a predator whose dynamics is forced by its prey, were the better‐ranking models (Table 4).

**Table 4:** Comparison of model selection criteria for MAR(1) and MAR(2) models with different structures. See Section 2 for definitions. The MAR(2) null + temperature uses April temperature with a 5‐year delay, which affects the predator only—this model adds temperature to the list of potential drivers for predator dynamics, as it was found marginally significant in previous MAR(1) analyses

Model type	LogLik.	AIC	AICc	BIC
MAR(1) null	−67.57	143.1	143.8	149.1
MAR(1) full	−64.41	140.8	142.3	149.8
MAR(2) full	−54.78	129.6	133.6	144.5
MAR(2) bottom‐up	−57.91	129.8	131.8	140.3
MAR(2) null	−58.78	129.6	131.0	138.5
MAR(2) null + temperature	−56.95	129.9	132.4	141.9

**Figure 2:**
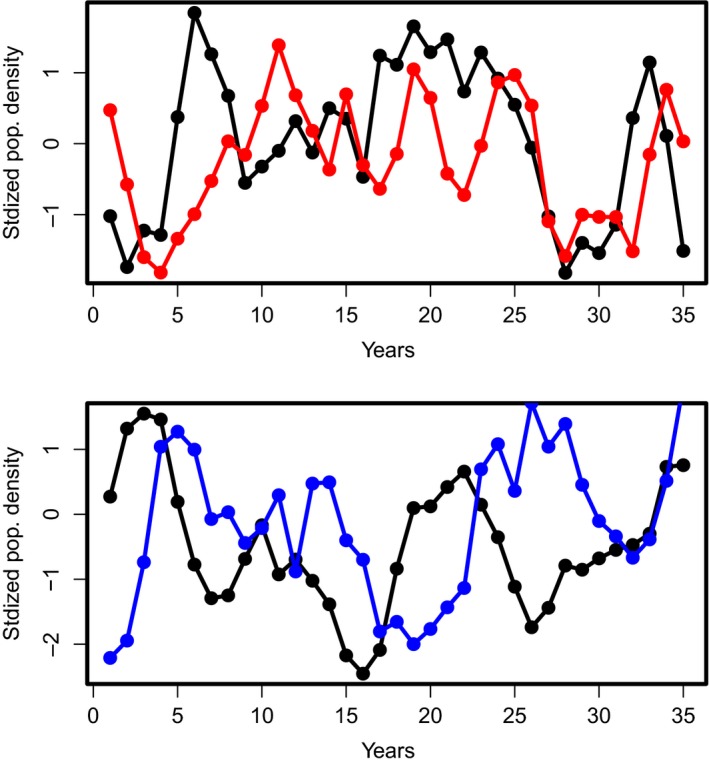
Time series of predator (gyrfalcon) and prey (rock ptarmigan) log‐densities, simulated for 35 years from the same starting conditions as the data, for the full MAR(1) model (top panel, predator in red) and the MAR(2) “bottom‐up’’ model (bottom panel, predator in blue)

Because AIC and BIC assess only one aspect of statistical model quality, the trade‐off between model parsimony and fit, we also present the results of simulations of the models (Figure 2). The examination of time series plots is, however, difficult because the simulated time series are relatively short and noisy.

**Figure 3:**
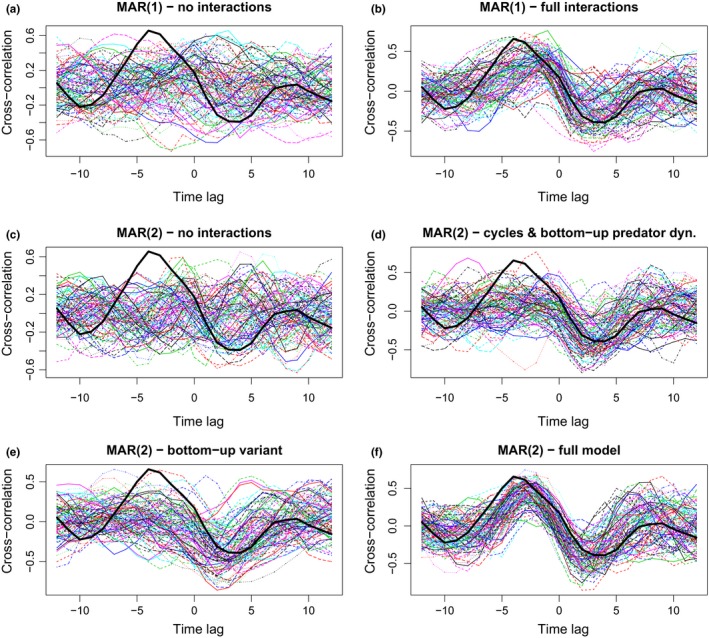
Cross‐correlation functions (CCFs) for the fitted models (A to F), defined as Cor(*x*_1,*t*+*k*_, *x*_2,*t*_) so that a maximum at *k* = −4 means that the predator time series *x*_2_ peaks on average 4 years after the prey *x*_1_. Each thin line corresponds to one simulation of the fitted model, within each panel. A and B show MAR(1) models, without and with interactions; while C to F show the CCFs of simulated MAR(2) models, without interactions (C), with only bottom‐up interactions (D), bottom‐up without delayed predator regulation (E), and (D) full MAR(2) model. The cross‐correlation for the real data is highlighted as a thick black line in all panels

We therefore simulated 100 datasets using the fitted models (Figure 3) and examined their cross‐correlations. These show that MAR(1) and MAR(2) models with reciprocal predator–prey feedback (full interaction matrices) outperform both the bottom‐up model (medium reproduction of the cross‐correlation pattern) and the null model (no reproduction of the cross‐correlation pattern).

### 3.3 Evaluating the quality of model identification from simulated data

We found that for a time series length *n* = 35, using a simulated MAR(1) full model with the fitted coefficients, we managed to recover this scenario around 50%–60% of all simulation runs (Table 5 below). By contrast, a simulated MAR(2) bottom‐up model was recovered >95% of the time for *n* = 35. However, percentages for both simulated models were all very close to 90% and 100% in the case of *n* = 100 (Table 5).

**Table 5:** Frequency of correct identification of MAR(1) full and MAR(2) bottom‐up models, for two time series lengths

Time series length	Simulated model	AIC	AICc	BIC
*n* = 35	MAR(1) full	0.52	0.56	0.64
	MAR(2) bottom‐up	0.98	0.98	0.97
*n* = 100	MAR(1) full	0.91	0.92	0.95
	MAR(2) bottom‐up	1	1	1

## 4 DISCUSSION

The percentage of explained variance in log‐abundances by the MAR(1) predator–prey model was about 60%; hence similar to the lynx–hare example of Vik et al. (2008). The full 2 × 2 interaction matrix in a MAR(1) framework provided a better model than a diagonal matrix, meaning there was causality (or *feedback*) between prey and predator dynamics in the sense of Granger (1969): The addition of the predator and prey variables reduced the residual variances of the time series models for the prey and predator, respectively.

Weather (i.e., April temperature 5 years lagged) was found to affect predator dynamics, revealing an influence of weather on gyrfalcon reproduction, which takes several years to impact the growth of the adult segment of the population. However, prey population growth was not affected by any of the weather covariates considered, neither in spring nor in winter (Supporting Information Appendix S2).

Before moving on to more ecological perspectives on the ptarmigan–gyrfalcon system, we would like to stress the importance of comprehensive model checking. Ecological time series are — by statistical standards — very short. If we had stopped the analyses to the MAR(1) model, which is customary in ecology (e.g., Hampton et al., 2013; Ives et al., 2003; Vik et al., 2008), we would have concluded unequivocally to a strong coupling between predator and prey (Table 2). However, identifying cycle causation requires to consider multiple potentially causal factors (see Barraquand et al., 2017, for a discussion).

Another reasonable hypothesis was that both species — the prey especially — could cycle independently (see e.g., Dobson & Hudson, 1992; for a host–parasite modeling study in a similar prey species). Contrasting top‐down versus bottom‐up dynamics required to formulate a MAR(*p*) model with *p* = 2 time lags (according to BIC, the optimal lag order was *p* = 2; *p* = 3 according to AIC). MAR(2) models were therefore found to realize a better trade‐off between parsimony and fit than MAR(1) models (Table 4). While the model with independently cycling populations fitted the data well, it produced unrealistic dynamics (i.e., no cross‐correlation, Figure 3). The bottom‐up predator–prey model, where the prey influences the predator but not the other way around, provided both a good fit and relatively realistic dynamics, though not as much as the models including reciprocal feedback (full‐matrix MAR(1) and MAR(2) models). The bottom‐up scenario could correspond, for example, to a case where the predator dynamics are driven by its prey, but prey dynamics are themselves driven by an interaction with a parasite (see Stenkewitz, Nielsen, Skírnisson, & Stefánsson, 2016, for an appreciation of host–parasite dynamics in Iceland rock ptarmigan). The bottom‐up scenario therefore fitted the data better in terms of trade‐off between parsimony and fit, but predicted the cross‐correlation pattern worse. Hence, both scenarios must be considered plausible. Further simulation results did help, however, to interpret better which scenario was the most likely given the data that have been collected.

This absence of strong conclusion on mechanisms, given the duration of the survey (34 years), may appear at first sight distressing to ecologists. But it is useful to keep in mind that, from the perspective of time series analyses, 34 points is very short. In fact, in his authoritative book on multivariate time series modeling, Lütkepohl (2005) shows that it can be hard to recover the simulated lag order of such simple MAR(1) and MAR(2) models. Specifically, Lütkepohl (2005) simulated a bivariate MAR(2) model with a time series length *n* = 30, fitted MAR(p) models up to order *p* = 6, and found only 32% of correctly classified simulations as *p* = 2 using AIC, with 42% classified as *p* = 1 (p. 155 in Lütkepohl, 2005). Using BIC, he found even 80% misclassified as MAR(1). Different model selection criteria gave different answers, and the BIC tended to be most conservative, but the baseline was that for *n* = 30, selection according to information criteria only gave inconsistent answers, while in most cases, the right model order was found for *n* = 100. Results were overall better with simulated bivariate MAR(1) models (p. 156 in Lütkepohl, 2005), where all model selection criteria were able to pinpoint the correct lag order at 90%. Such results, however, are likely to be model‐structure and model‐parameter specific (they depend on MAR coefficient values); therefore, we performed again such analysis for the models that we fitted (Section 2.4). With the current length of our dataset *n* = 34, the important message from our simulations is that based on AIC or BIC, we are much more likely to mistake a fully interacting predator–prey system for a bottom‐up system than the reverse.

From our simulation experiments, we can derive three lessons. First, from an ecological viewpoint, given that the full interaction MAR(1) model both predicts the cross‐correlation pattern better and is the most likely to be misidentified as MAR(2) bottom‐up, we should not give too much weight to the better (lower) AIC and BIC scores of the MAR(2) bottom‐up model. It is more likely that top‐down prey regulation and therefore reciprocal predator–prey feedback is at work here. Second, from a more statistical viewpoint, it is informative to notice that whether MAR(1) or MAR(2) models are better identified is parameter‐specific: Sometimes a MAR(1) model will be more likely to be correctly classified (as in the simulation study of Lütkepohl, 2005), sometimes a MAR(2) will (our case study). The corollary being that new simulations from MAR(p) or other time series models will be required for each new ecological case study, in order to see which scenarios are the most likely to be misidentified. Third, we found, in agreement with Lütkepohl (2005), that time series of around 100 points are needed to allow for fairly reliable inference of top‐down versus bottom‐up dynamics in systems of 2 cyclic species (less points might be required for species with simpler dynamics).

Given that the data presented here are collected once a year for the most part, and that it is not feasible to census the population much more frequently with current means (other technologies would be necessary, such as camera traps or DNA‐based evidence), it is unlikely that we will get the time series near 100 years within acceptable time frames for management of both populations (i.e., conservation of the gyrfalcon and sustainable hunting management of ptarmigan). Therefore, differentiating unequivocally between the bottom‐up and predator–prey feedback scenarios will likely require other type of models and data. We still view the MAR(p) approach as useful, however, as a means to delineate likely scenarios to investigate further, and check for important abiotic drivers that need to be considered. Why the ptarmigan population growth is not affected — at the NE Iceland scale — by weather variables is also a puzzling question for further study, though there are other instances of this absence of weather effects (Wann, Aldridge, & Braun, 2016).

Mechanistic modeling might help to understand further the effect of drivers on ptarmigan dynamics during certain phases of the cycle. For instance, are ptarmigan declines mainly driven by its predator or mainly by other causes such as parasites (e.g., Dobson & Hudson, 1992)? Rough estimates of the required predation demonstrate why this question is intrinsically difficult. Around 100 adult predator pairs can be found near peak abundance on the NE Iceland ptarmigan management zone and to these correspond about 100,000 ptarmigan individuals at best (Sturludottir, Nielsen, & Stefansson, 2018). One might think, given these numbers, that the predators are unlikely to make their prey decline. Ptarmigan, however, have a slow, long‐period cycle (Figure 1). Therefore, they decrease at worst by ≈  20,000 in a single year. A quick division indicates that about 200 would have to be eaten during the year by a predator pair for such a decrease to occur — assuming, as a first approximation, that increases in the ptarmigan population due to reproduction are offset by other causes of death than predation. This quantity, 200 kills a year, is an order of magnitude that represents a fairly high yet doable consumption by a predator pair. This might be tested further by fitting more mechanistic predator–prey models. We note in passing that the tight interaction between predator and prey here might not be typical of all gyrfalcon–ptarmigan populations (Nielsen, 2011). Outside Iceland, other prey and predators may come into play and influence both species dynamics (e.g., rodents in particular can have a strong influence on both the birds of prey and the ground‐breeding birds, Angelstam, Lindström, & Widén, 1984; Barraquand, New, Redpath, & Matthiopoulos, 2015). Studies on similar birds of prey in continental areas have typically showed weaker predator–prey coupling and more pronounced weather effects (Tornberg et al., 2013).

Although we currently do not possess all the information necessary to parameterize mechanistic predator–prey or host–parasite models, we suggest a few directions based on our results: We recommend to focus on measuring parameters related to top‐down control or that could explain independent cycling of the prey (rather than weather effects on vital rates, unless the spatial dimension is considered). First, regarding top‐down control, we have a rather imperfect knowledge of the predator population, especially its non‐territorial segment (Nielsen, 2011). Non‐territorial floaters can indeed be rather numerous in both real raptor populations (Katzner, Ivy, Bragin, Milner‐Gulland, & DeWoody, 2011) and parameterized bird population models (Barraquand et al., 2014). Floater numbers could therefore change our perception of predator impacts on prey dynamics (i.e., the predator population might increase by half or more). Demographic modeling of the predator population and its various life stages is therefore in order — we are currently examining CMR data and hoping for DNA‐based bird identification. Second, as we could not definitely reject the bottom‐up hypothesis, the host–parasite hypothesis for the ptarmigan dynamics (see Stenkewitz et al., 2016) needs to be examined. We therefore have to know more about parasite loads and their potential impact on ptarmigan population growth. Third, there are spatial aspects in the dynamics of gyrfalcon and ptarmigan that we have not tackled. It is plausible, for instance, that the weather does not affect ptarmigan growth at the scale of NE Iceland, using population‐level variables, and yet that weather locally affects the survival of ptarmigan chicks, as additional data seem to suggest: Nielsen et al. (2004) found that mean wind speed and mean precipitation in June–July explained a considerable part of the variance in chick production.

## 5 CONCLUSION

Using long time series by ecological standards (34 years) but short ones by statistical standards, we found evidence of reciprocal predator–prey feedback in this cyclic predator–prey system, without being able to exclude definitely more bottom‐up predator–prey dynamics. MAR(*p*) models with *p* = 1,2 described well this system as a forced oscillator, although the unexplained noise was generally stronger than weather effects, which may point to other important biotic factors driving the dynamics, such as parasites. Simulations of the fitted models revealed than unequivocal inference of bottom‐up versus reciprocal predator–prey coupling (i.e., including top‐down predator influence on prey) would require about a century of time series data. Our results have therefore implications for other studies on vertebrates with relatively slow life histories (compared to, e.g., plankton sampled many times a year). We think that additional demographic data (e.g., through capture–recapture, genetics,…) should always be considered in conjunction to counts taken once or twice a year, if one of the goals of a vertebrate monitoring study is to infer interactions between the populations of different species.

## AUTHOR CONTRIBUTIONS

FB and ÓKN conceived the ideas; ÓKN collected the biological data and identified relevant abiotic variables; FB designed the models and analyzed the data; FB led the writing of the manuscript with inputs from ÓKN. Both authors contributed critically to the subsequent drafts and gave final approval for publication.

## DATA ACCESSIBILITY

Data and computer codes for analyses are available in GitHub repository: https://github.com/fbarraquand/GyrfalconPtarmigan_MAR and have been archived at Zenodo with https://doi.org/10.5281/zenodo.1411871.
