# Corrigendum: Predator‐prey feedback in a gyrfalcon‐ptarmigan system?

**DOI:** 10.1002/ece3.6336

**Published:** 2020-07-24

**Authors:** Frédéric Barraquand, Ólafur K. Nielsen

**Affiliations:** ^1^ CNRS Institute of Mathematics of Bordeaux Talence France; ^2^ Integrative and Theoretical Ecology LabEx COTE University of Bordeaux Pessac France; ^3^ Icelandic Institute of Natural History Garðabær Iceland

In our article on gyrfalcon and ptarmigan population cycles in Iceland (Barraquand & Nielsen, 2018), we contrasted several models of predator–prey dynamics, including models with reciprocal predator–prey interactions (with and without abiotic covariates), models with independent predator and prey dynamics, as well as an in‐between dubbed “bottom‐up,” where prey density cycles independently and predator growth benefits from increases in prey density, but predator density exerts no top‐down control in return. The main conclusion was that reciprocal effects of predator and prey densities on their population growth rates—that is, predator–prey feedback—were likely and that although fitting well, models for independent cyclic dynamics in predator and prey produced unrealistic cross‐correlation patterns. However, the bottom‐up scenario, where the prey population cycles independently of its predator—which follows the cycle without causing it—could not be excluded.

Unfortunately, while the bottom‐up model was correctly written in the main text, it was incorrectly implemented into code. In fact, the incorrectly coded model included a nonzero predator → prey link $b_{12}^{\left(2\right)}$ in matrix **B** ^(2)^ instead of a nonzero prey → predator link $b_{21}^{\left(2\right)}$ . The bottom‐up model is the only model affected by this error. We have corrected the code^1^ and deposited a new version at Zenodo http://doi.org/10.5281/zenodo.3740457. After implementing these corrections, we have noticed that:

- The bottom‐up model ranks slightly better in terms of model selection criteria (AIC: 125.4, AICc: 127.3, BIC: 135.9). It therefore becomes the best scoring model. However, the differences in scores are small and should not be overinterpreted.
- The cross‐correlation patterns produced by the true bottom‐up model are also more realistic, as indicated by the corrected Figure 3 below, and become difficult to distinguish from those of full‐interaction MAR(1) and MAR(2) models (modeling predator–prey feedback).
- The identifiability properties of the bottom‐up MAR(2) model and the full‐interaction MAR(1) model do not change (i.e., Table 5 remains similar): with *n* = 35, a bottom‐up simulation has >95% chances to be identified as such but a MAR(1) top‐down model has only ≈1/2 chances to be identified as such and 1/2 to be mistaken for a bottom‐up MAR(2).

**FIGURE 3:**
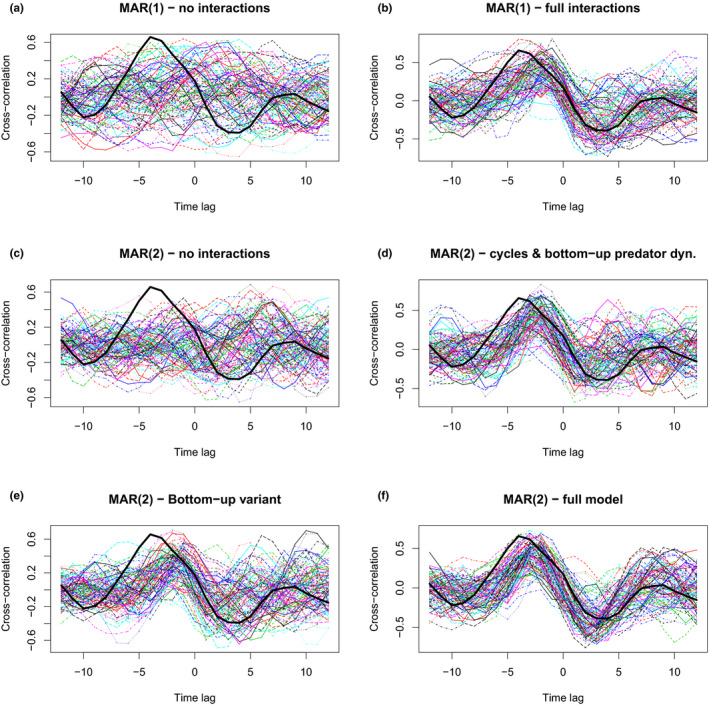
Cross‐correlation functions (CCFs) for the fitted models (a to f), defined as $\text{Cor}\left(x_{1,t+k},x_{2,t}\right)$ so that a maximum at k=-4 means that the predator time series $x_{2}$ peaks on average 4 years after the prey $x_{1}$ . Each thin line corresponds to one simulation of the fitted model, within each panel. Panels (a) and (b) show MAR(1) models, without and with interactions, while (c) to (f) show the CCFs of simulated MAR(2) models, without interactions (c), with only bottom‐up interactions (d), bottom‐up with direct prey effect (e), and (f) full MAR(2) model. The cross‐correlation for the real data is highlighted as a thick black line in all panels.

We considered in Figure 3 a bottom‐up model variant where $b_{22}^{\left(2\right)}=0$ . Upon close re‐examination after implementing the required correction, this model variant was found to behave appropriately (stable distribution of predator and prey densities) when $b_{22}^{\left(2\right)}$ is set to zero after model fitting but not when the model is fitted with $b_{22}^{\left(2\right)}=0$ a priori. Hence, we have considered for the corrected Figure 3 a new model bottom‐up variant with matrices

$$B^{\left(1\right)}=\left(\begin{array}{cc} b_{11}^{\left(1\right)} & 0\\ b_{21}^{\left(1\right)} & b_{22}^{\left(1\right)} \end{array}\right)$$

and

$$B^{\left(2\right)}=\left(\begin{array}{cc} b_{11}^{\left(2\right)} & 0\\ 0 & b_{22}^{\left(2\right)} \end{array}\right)$$

This models a more immediate effect of prey density; the original model favored a more delayed effect $b_{21}^{\left(2\right)}$ , due to the fact that we model reproductive predator densities. The revised bottom‐up model variant has similar cross‐correlation properties, yet fits slightly less well than the (corrected) original bottom‐up model given that the number of parameters is identical (AIC: 128.9, AICc: 130.9, BIC: 139.4).

We previously stated that we found evidence of predator–prey feedback in gyrfalcon‐ptarmigan, as opposed to independent dynamics, without being able to exclude bottom‐up dynamics. Overall these results still stand, though more weight should be given to the bottom‐up hypothesis—perhaps an equal weight to that of the predator–prey feedback hypothesis. While the bottom‐up model realizes the best trade‐off between parsimony and fit, a true predator–prey reciprocal interaction could have as high as a 50% chance to be mistaken for the bottom‐up model with *n* = 35 years. Simulations with larger sample sizes, using the corrected code, still show that unequivocal inference of MAR(2) bottom‐up versus MAR(1) predator–prey feedback would require about a century of annually sampled predator and prey densities. Deciding between the two hypotheses will likely require additional demographic and trophic data.

## ENDNOTE

^1^

https://github.com/fbarraquand/GyrfalconPtarmigan_MAR
