# Supplementary material for: Predator‐prey feedback in a gyrfalcon‐ptarmigan system?
Source: Ecol Evol. 2018 Nov 28;8(24):12425–34. doi: 10.1002/ece3.4563 (PMC6308892; doi:10.1002/ece3.4563)
Supplement: Supplementary file 1 [file ECE3-8-12425-s001.pdf]

Supporting Information for Barraquand, F. & Nielsen, Ó. K., 2018. Predator-prey feedback in a gyrfalcon-ptarmigan system? *Ecology and Evolution*

Supporting Information S1 - Alternative model fitting

Parameters	Meaning	Point estimate	LB 95%	HB 95%
$b_{11} - 1$	prey $\rightarrow$ prey	-0.24	-0.47	-0.03
b_{12}	predator $\rightarrow$ prey	-0.24	-0.47	-0.04
b_{21}	prey $\rightarrow$ predator	0.23	0.04	0.46
$b_{22} - 1$	predator $\rightarrow$ predator	-0.34	-0.57	-0.12
σ_1	noise species 1	0.67	0.53	0.87
σ_2	noise species 2	0.66	0.52	0.85

Table S1.1: Estimates of the full MAR(1) model using JAGS, for comparison with the results of the main text.

Supporting Information S2 - Effects of winter weather

We tested the effect of winter weather by introducing new winter weather variables into the MAR(1) models:

- The mean winter temperature from December to March
- The average of log(precipitation) over the same period

We also considered minimum temperature but this did not alter the following results.

The two above mentioned winter weather variables were inserted in place of spring weather variables for ptarmigan into a MAR(1) model. The estimated parameters are reproduced in Table S2.1 and the Information Criteria, with previous models for comparison, in Table S2.2. None of the models are able to significantly improve the fit, although it is possible that a weakly statistically significant effect of winter temperature exists.

Parameter	value	SE	low 95% CI	up 95% CI
b_{11}	0.6837	0.1169	0.4546	0.9129
b_{21}	0.2035	0.1045	-0.0013	0.4084
b_{12}	-0.1999	0.1077	-0.4111	0.0113
b_{22}	0.7018	0.1044	0.4971	0.9065
Mean winter temp$_{t+1}$	*-0.1466*	*0.1210*	*-0.3838*	*0.0906*
Winter precipitation$_{t+1}$	*-0.1720*	*0.1185*	*-0.4044*	*0.0602*
temperatureApril$_{t-4}$	0.2071	0.1062	-0.0010	0.4153
rainApril$_{t-4}$	-0.0557	0.1068	-0.2652	0.1537
σ_1^2	0.3635	0.0742	0.2092	0.5602
σ_2^2	0.3422	0.0734	0.1945	0.5314

Table S2.1: Coefficients for biotic and abiotic effects on population growth. Species 1 is ptarmigan and species 2 gyrfalcon. Winter variables only affect species 1 while April variables, delayed by 5 years (we model the effect of variables at $t-4$ on growth between t and $t+1$), affect only species 2's population growth. *Effects of winter variables are depicted in italics.*

Model type	logLik.	AIC	AICc	BIC
MAR(1) null	-70.01	148.0	148.7	154.1
MAR(1) full	-66.14	144.3	145.7	153.4
MAR(1) full + May temperature year t	-63.98	144.0	146.4	156.2
MAR(1) null + May temperature year t	-67.03	146.1	147.4	155.2
MAR(1) full + May temperature of year $t-1$	-63.94	143.9	146.3	156.1
MAR(1) full + May log(precipitation) of $t-1$	-64.89	145.8	148.2	158.0
MAR(1) full + May temp + log(precipitation)	-62.81	145.6	149.5	160.9
MAR(1) full + Mean Winter temp + mean log(precipitation)	-62.72	145.4	149.3	160.7
MAR(1) full + Min Winter temp + mean log(precipitation)	-62.72	145.4	149.3	160.7

Table S2.2: Comparison of model selection criteria for MAR(1) models. MAR(1) 'null' indicates a diagonal $\mathbf{B}$ matrix while MAR(1) 'full' indicates a full 2×2 interaction matrix. Models including temperature (third row and below) effects on growth rates take the form $\mathbf{x}_{t+1} = \mathbf{a} + \mathbf{B}\mathbf{x}_t + \mathbf{C}\mathbf{u}_t + \mathbf{e}_t, \mathbf{e}_t \sim \mathcal{N}_2(0, \mathbf{\Sigma})$. Here the environmental vector $\mathbf{u}_t = (T_{t-l_P}, R_{t-l_P}, T_{t-l_G}, R_{t-l_G})'$, with T the temperature and R log-rainfall. There is a timelag l_P for the ptarmigan (0 or 1 year, depending on the month) and $l_G = 5$ for the gyrfalcon. IC scores for the two winter models are depicted on the last two rows.
